# Honey bees respond to multimodal stimuli following the principle of inverse effectiveness

**DOI:** 10.1242/jeb.243832

**Published:** 2022-05-24

**Authors:** Oswaldo Gil-Guevara, Hernan A. Bernal, Andre J. Riveros

**Affiliations:** 1 Departamento de Biología, Facultad de Ciencias Naturales, Universidad del Rosario, Cra. 26 #63B-48, Bogotá, Colombia; 2 Programa de Ingeniería Biomédica, Escuela de Medicina y Ciencias de la Salud, Universidad del Rosario, Bogotá, Colombia; 3 Department of Neuroscience, University of Arizona, Tucson, AZ 85721, USA

**Keywords:** Multimodal integration, Bimodal signals, PER, Associative learning, Cross-modal integration, *Apis mellifera*, Absolute conditioning

## Abstract

Multisensory integration is assumed to entail benefits for receivers across multiple ecological contexts. However, signal integration effectiveness is constrained by features of the spatiotemporal and intensity domains. How sensory modalities are integrated during tasks facilitated by learning and memory, such as pollination, remains unsolved. Honey bees use olfactory and visual cues during foraging, making them a good model to study the use of multimodal signals. Here, we examined the effect of stimulus intensity on both learning and memory performance of bees trained using unimodal or bimodal stimuli. We measured the performance and the latency response across planned discrete levels of stimulus intensity. We employed the conditioning of the proboscis extension response protocol in honey bees using an electromechanical setup allowing us to control simultaneously and precisely olfactory and visual stimuli at different intensities. Our results show that the bimodal enhancement during learning and memory was higher as the intensity decreased when the separate individual components were least effective. Still, this effect was not detectable for the latency of response. Remarkably, these results support the principle of inverse effectiveness, traditionally studied in vertebrates, predicting that multisensory stimuli are more effectively integrated when the best unisensory response is relatively weak. Thus, we argue that the performance of the bees while using a bimodal stimulus depends on the interaction and intensity of its individual components. We further hold that the inclusion of findings across all levels of analysis enriches the traditional understanding of the mechanics and reliance of complex signals in honey bees.

## Abstract (precis)

**Summary:** Bimodal enhancement during learning and memory tasks in africanized honey bees increases as the stimulus intensity of its unimodal components decreases; this indicates that learning performance depends on the interaction between the intensity of its components and the nature of the sensory modalities involved, supporting the principle of inverse effectiveness.

## INTRODUCTION

The integration of information from multiple sensory modalities enables a more precise representation of the environment and often increases behavioral performance (Ghosh et al., 2017; Hartline et al., 1978; Meredith and Stein, 1983; Shams and Seitz, 2008; Stein, 1998; Zahar et al., 2009). While multimodal integration occurs across diverse animal taxons and behavioral contexts, the extent of the behavioral enhancements elicited by multimodal signals depends on key physical factors of the compound stimuli (Cappe et al., 2010; Chandrasekaran, 2017; Otto et al., 2013; Stein and Stanford, 2008). Signal intensity, a conspicuous feature of signals, has rarely been evaluated in behavioral contexts where learning a multimodal signal has a clear adaptive value. Multisensory integration is especially relevant in tasks requiring learning and memory of signals such as those used in the interactions between pollinators and plants (Kulahci et al., 2008; Leonard and Masek, 2014). At an adaptive level, the multi-component flower traits (color, pattern, shape and scent) may reduce pollinator uncertainty by increasing the amount of information (Chittka and Thomson, 2001; Leonard and Francis, 2017; Leonard et al., 2011a,b). At the proximate level (a non-mutually exclusive perspective), the physical properties such as spatiotemporal and salience aspects of signals may affect the effectiveness of multimodal signals (Meredith and Stein, 1983; Otto et al., 2013; Riveros et al., 2020; Rubi and Stephens, 2016a; Stein et al., 1988). In particular, deciphering the effects of intensity variation of the multicomponent signal elements and their possible interactions is needed. Despite this, the effect of varying intensity levels during multimodal learning is still unclear.

Signal detection and discrimination in noisy backgrounds is a universal problem in sensory processing (Babineau et al., 2007) and different species rely on different strategies to tackle the challenge; the use of multiple modalities is one such tactic (Wiley, 2006). Multimodal information occurs across a wide range of ecologically relevant tasks (perception, locomotion, communication) (Cappe et al., 2012; Cowan and Fortune, 2007; de Luna et al., 2010; Narins et al., 2003). The omnipresence of multimodal signals fosters the idea that an enhancement of the receiver's performance is the main benefit of integrating multiple sources of information (Kulahci et al., 2008; Partan, 2017; Siddall and Marples, 2008). Here, the senders gain from higher signal conspicuousness and redundancy, and the receivers benefit from enhanced learning and memory, attention and overall information processing (Akre and Ryan, 2010; Arak and Enquist, 1993; Balkenius and Balkenius, 2016; Hebets and Papaj, 2005; Redhead, 2007; Rubi and Stephens, 2016a; Sutherland and Mackintosh, 1971; ten Cate and Rowe, 2007). The cognitive basis of the perception of the diverse floral displays offered by plants is also dependent on the innate and learned behavioral responses of pollinators (Schiestl and Johnson, 2013). As a consequence, the increased amount of information derived from multiple floral traits should facilitate discrimination and learning, enhancing foraging efficiency (Kulahci et al., 2008; Leonard et al., 2011a,b). For a floral diurnal visitor, visual and olfactory elements are the most conspicuous components of the signal and determine the initial contact (Raguso, 2004). It has been suggested that even some nocturnal bees rely on both olfaction and vision to exploit flower resources (Liporoni et al., 2020). In short, it has been hypothesized that multimodal signals might boost pollinators' attention towards a particular floral display, as a result of increased conspicuousness (Leonard et al., 2011b). Therefore, a potential enhancement during learning and memory is also predicted (Leonard and Francis, 2017; Leonard et al., 2011b). Importantly, previous studies have detected the modulation of one modality by the learning of another, suggesting the existence of interactions during the acquisition of bimodal elements, at the neural level (Giurfa, 2003; Mota et al., 2011; Sandoz, 2011).

Multimodal signals might be more beneficial than unimodal ones, either because they provide a higher amount of information or because they facilitate receiver perception a pair of non-mutually exclusive hypotheses (Rubi and Stephens, 2016b). From the information theory point of view, however, multimodal signals might not necessarily be better than unimodal ones; that is, in some instances, receivers perform equally well facing unimodal or multimodal signals (Rubi and Stephens, 2016a; Wilson et al., 2013). This reinforces the idea that multimodal signals enable performance enhancements mainly at the signal processing level (Rubi and Stephens, 2016a,b). Also, recent studies have not found the expected differences in performance between bimodal and unimodal stimuli (Riveros et al., 2020; Rubi and Stephens, 2016b). To understand how multimodal signals might benefit receivers, a direct comparison between the effects of unimodal and multimodal signals is required. Such comparisons should avoid confounding the effects of multiple components with multiple modalities or the inappropriate distinction between innate and learned responses (Rubi and Stephens, 2016b). It is also necessary to consider the physical properties of the stimuli (i.e. synchrony and intensity level). In addition, bumble bees do not necessarily enhance their performance when trained using bimodal versus unimodal stimuli under restrained conditions (Riveros et al., 2020), which contrasts with the performance of bees in free flight (Kulahci et al., 2008). However, it is not clear whether the discrepancy derives from differences intrinsic to the methods, such as synchrony/asynchrony in the presentation of components or perceived variation in olfactory and visual stimuli intensity (Riveros et al., 2020).

The honey bee has been used to study the functional mechanisms of learning and memory (Giurfa, 2003; MaBouDi et al., 2017; Matsumoto et al., 2012; Takeda, 1961). Most of the attention has historically focused on anatomical, neuronal and behavioral aspects of unisensory olfaction (Carcaud et al., 2018; MaBouDi et al., 2017; Mauelshagen, 1993; Riveros and Gronenberg, 2012; Sandoz, 2011) or vision (Ehmer and Gronenberg, 2002; Horridge, 2009; Jernigan et al., 2014; Riveros and Gronenberg, 2012). Multisensory integration of bees during learning has received less attention, with some significant accounts (Gerber and Smith, 1998; Kulahci et al., 2008; Leonard et al., 2011a,b,c; Mota et al., 2011; Riveros et al., 2020; Strube-Bloss and Rössler, 2018). Although the role of intensity thresholds has been extensively examined both in vision (Avarguès-Weber and Giurfa, 2014; Backhaus, 1991; Chittka, 1992; Hempel De Ibarra et al., 2000; Katzenberger et al., 2013; Neumeyer, 1981; Nouvian and Galizia, 2020) and olfaction (Bhagavan and Smith, 1997; Wright and Smith, 2004; Wright et al., 2002, 2005), the intensity variation during multimodal learning and memory tasks has rarely been directly explored (but see Katzenberger et al., 2013).

Typically, and across contexts, an animal’s response increases together with the intensity of the stimulus (Bhagavan and Smith, 1997; Gil-Guevara and Amézquita, 2020; Hempel De Ibarra et al., 2000; Mackintosh, 1974; Wright et al., 2005). Similarly, the higher the intensity, the stronger the association between a stimulus and a reward, following associative learning model predictions (Rescorla and Wagner, 1972). In nature, several factors influence the intensity of individual floral components, mainly during signal production and transmission (Bradbury and Vehrencamp, 1998). Importantly, at the perceptual level, the so-called principles of multisensory integration compare the effectiveness of a bimodal stimulus relative to its unimodal components (Meredith and Stein, 1983; Otto et al., 2013; Stein and Stanford, 2008; Stein et al., 1988). Unimodal components are effectively integrated (thus, increasing the strength of the multisensory response) when they originate from the same place, when they occur synchronously and when the individual unisensory responses are weak as a result of signal intensity variation (Meredith and Stein, 1986; Stanford and Stein, 2007; Stein et al., 1988). In field conditions, these physical properties of stimuli (location, timing and intensity) determining the extent of the integration may interact with other components of the signal, affecting pollinator performance (Hebets and Papaj, 2005; Leonard and Masek, 2014).

To study the effect of intensity levels during multimodal learning, precise control of the stimuli presented to individuals is therefore required, a difficult task when using free-flight protocols where salience and synchrony vary depending on the particular flight pattern (speed, angle, etc.) (Leonard and Masek, 2014; Riveros et al., 2020; Wright et al., 2009). Alternatively, the conditioning of the proboscis extension response (PER) protocol (Giurfa and Sandoz, 2012; Matsumoto et al., 2012), where bees are tested under restrained conditions, enables a more precise stimulus delivery (Leonard and Masek, 2014). The PER is a natural appetitive response where bees extend the proboscis upon sensory stimulation (antennae, tarsi) with a sweet substance (floral nectar) (Bitterman et al., 1983; Takeda, 1961). During a training experiment, the PER is conditioned by pairing the presentation of the unconditioned stimulus (US; sucrose solution) with a conditioned stimulus (CS; here, odor/color). After several repeated pairings, a response to the CS eliciting a PER in the absence of the US (the CS now serves as a predictor of the US) is considered as a proxy of learning. This protocol has been historically used to examine learning and memory capabilities (Giurfa and Sandoz, 2012).

In this study, we aimed to explore the possible interactions between sensory modalities and intensities during a learning task. We evaluated the learning performance of honey bees under restrained conditions (PER) by comparing the effect of variation in the intensity components between bimodal and unimodal stimuli. We relied on Africanized honey bees (*Apis mellifera scutellata*) as they can be readily trained to both olfactory and visual stimuli (Jernigan et al., 2014). We tested whether an enhancement in learning and memory performance results from bimodal stimulation relative to unimodal signals and whether latency of the response is affected. We constructed this hypothesis based on the idea that a compound signal should provide redundant information, eliciting better learning (Mackintosh, 1974). Here, redundant signals possess multiple components that improve the accuracy of information transmission (Hebets and Papaj, 2005; Leonard et al., 2011c) and hence, from the receptor point of view, might lead to a stronger association between the compound signal and its message than is possible in the case of unimodal signals (Mackintosh, 1974; Rowe, 1999). We also tested whether increasing intensity enhances learning performance when trained using unimodal (olfactory/visual) and bimodal stimuli. This last hypothesis allowed us to test a cross-modal phenomenon, known as the ‘principle of inverse effectiveness’ previously detected in some mammals, in which the lower the effectiveness of the individual components of a multimodal stimulus, the higher the relative performance when combined (Holmes, 2009; Otto et al., 2013; Stein and Stanford, 2008). That is, we tested whether a bimodal stimulus composed of low intensity units results in a higher performance relative to that produced by the unimodal stimuli of the same intensity. The principle of inverse effectiveness is part of a set of conceptually simple rules that predict when multisensory integration is physiologically more strong, efficient or prevalent. These rules suggest that several unimodal stimuli are more likely to be integrated as a single compound when presented from the same location (spatial rule) or the same temporal window (temporal rule), or when the unisensory components are relatively weak (principle of inverse effectiveness) (Chandrasekaran, 2017; Guo and Guo, 2005; Stein and Stanford, 2008). Under this framework, the principle of inverse effectiveness would make behavioral sense if unimodal sensory stimuli are sufficient to solve learning tasks when presented at high intensities, but would be insufficient at low intensities, being surpassed by the learning induced by bimodal stimulation at the same intensities (Guo and Guo, 2005; Stein and Stanford, 2008; Stein et al., 1988). Finally, we tested the effect of intensity levels within each modality (olfactory, visual and bimodal) across intensities. Using the PER conditioning protocol, we examined how the learning ability of bees is affected by intensity within different modalities.

We found that bees trained using a bimodal stimulus did not necessarily exhibit the highest performance. During bimodal learning and memory tasks, the greatest enhancement in performance was achieved when the signal components consisted of low intensity stimuli. However, this relative bimodal enhancement was not observed in bees trained with stimuli of mid and high intensity. Similar trends were followed when evaluating memory retention. Also, we found that the latency of response during bimodal learning was not affected by the variation in stimuli intensity and was only affected by the modality type. Our results suggest that the complex interactions between modalities during multimodal learning can be modulated by the intensity level.

## MATERIALS AND METHODS

### Bee collection and maintenance

Africanized honey bees, *Apis mellifera scutellata* Lepeletier 1836, were obtained from hives maintained at the Universidad Nacional de Colombia in Bogota (4.642419N, −74.081839W; ∼2600 m elevation; annual average climatic conditions: relative humidity, RH: 80–85%; temperature: 14.2−19.7°C, ±8.4°C). Worker bees leaving the hive were collected (13:00 h–16:00 h) using a pyramidal translucent acrylic trap (Matsumoto et al., 2012). Then, honeybees were ice anesthetized (Jernigan et al., 2014) and harnessed into custom 3D printed plastic tubes. After recovery, bees were fed *ad libitum* with sucrose–water (50% w/w) and maintained overnight in a polypropylene box with a window that enabled natural illumination (aiming to maintain photoperiod) and humidity stability (58% RH). The next morning, the bees were tested for motivation using the PER elicited by antennal stimulation with the sucrose solution (50% w/w). Only those individuals responding were included in experiments. At the end of both the training and memory retention tests, all surviving bees were released. To avoid using a bee more than once, we labelled them with a small drop of enamel paint on the dorsal surface of the thorax before release.

### Training apparatus

**Fig. 1.**
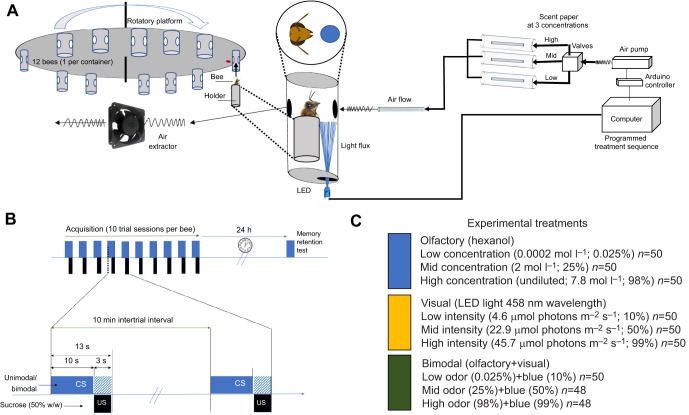
**Conditioning of restrained bees to olfactory, visual and bimodal stimuli.** (A). Schematic diagram of the electromechanical set up to achieve precise control of the light intensity, odor concentration and timing of delivery of unimodal and bimodal stimuli. A pre-programmed sequence of visual and olfactory stimuli, loaded on a PC, implemented custom software to direct an Arduino Uno microcontroller. The instructions triggered a set of electro-valves allowing different concentrations of 1-hexanol and different intensities of light intensity (LED) to be delivered to individual honeybees. (B) A classical conditioning protocol under the proboscis extension response (PER) paradigm, allowed the training of 12 bees harnessed on a rotatory platform per session. The experimental procedure for absolute conditioning (unimodal conditioned stimulus, CS: olfactory or visual; bimodal CS: olfactory and visual). Bees were conditioned using 10 trials. The conditioning sequence consisted of 10 s of stimulation followed by 3 s of paired (shaded areas) CS and unconditioned stimulus (US: 50% w/w sucrose solution). Then, an inter-trial interval of 10 min without stimulation followed for each bee before they were subjected to the subsequent trial. This procedure was repeated until 10 trials had been completed for all 12 bees. A memory retention test on the particular stimuli (unimodal or bimodal) was performed 24 h later without the US. Binary behavioral responses (PER) and latency (s) to PER were registered. (C) Description of the experimental treatments and the final sample sizes achieved per level of treatment during the experiments.

We adapted a training apparatus that allows both precise and automatic delivery of olfactory and visual stimuli (Fig. 1A) (Jernigan et al., 2014; Riveros and Gronenberg, 2009; Riveros et al., 2020). The setup included 12 individual chambers coated with aluminium foil tape to homogenize the reflectance of light emitted from a LED located at the bottom of the compartment (see Fig. 1A). Each chamber was attached to an acrylic rotatory platform (diameter 0.52 m) and had two openings – one in the front and in one the back – enabling a stream of pumped air to flow through the chamber and access for the experimenter to provide the reward. As each chamber contained an individual harnessed bee, we trained 12 bees at a time.

For stimulus delivery, a sequence of instructions with different concentrations and intensities was programmed in advance. An *Arduino Uno* microcontroller (v. REV 3 SMD) using custom code implemented in *Arduino* (v. 1.8.7) on a PC running *Processing* software (v. 3.5.3) (Reas and Fry, 2014) read and executed the stimulus sequence. This system controlled the air flow provided by a pipe system connecting an air pump with a set of parallel electronic valves that allowed the air to flow into a set of three parallel glass tubes containing filter paper with a scent. The air flow reached the chamber at a volume of 1.08 l min^−1^ (Fluke VT Plus HF gas flow analyzer) after mixing with a parallel constant airflow of clean air (0.33 l min^−1^) aimed to reduce the possibility that bees learned the mechanical stimulation by air. Finally, the odor airflow was effectively cleaned out by an air extractor in the back of the chamber before and after each odor stimulation (0.30 l min^−1^) (Fig. 1A). Simultaneously, our system controlled light intensity by automatically varying the electric current reaching the LED. In addition to controlling stimulus delivery, our software code also allowed recording of the timing of behavioral events (latency of response, see below) with a built-in synchronized chronometer. Because of the restraining method, the lower portion of the bee eye may have received direct light, while other regions received diffuse light; however, we did not measure light distribution inside the chamber (Jernigan et al., 2014). We applied 10 μl of the scent solution at the corresponding concentration (see ‘Training stimuli’, below) to a piece of filter paper (∼10×4 mm) and placed it in the respective glass tube of the training device (see Fig. 1A). We replaced the filter paper with the solution concentration after 3 consecutive puffs during the training trials of each training session.

### Training stimuli

**Fig. 2.**
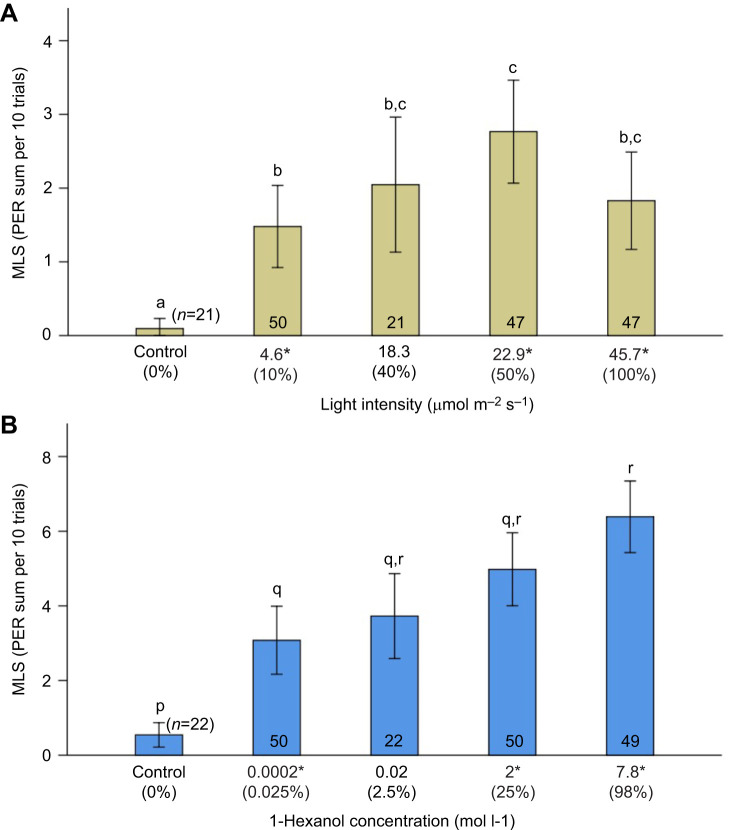
**Minimum thresholds for visual and olfactory learning.** (A) Five light intensities were tested (from 0 to 45.7 µmol photos m^−2^ s^−1^, the maximum current supported by the LED). The median learning score (MLS) in response to the lowest intensity tested (4.6 µmol photos m^−2^ s^−1^; 10% of maximum intensity) was significantly different from that for the medium intensity (22.9 µmol photos m^−2^ s^−1^; 50%) and control (0%; see Materials and Methods). (B) Five concentrations of 1-hexanol (from 0 to 7.8 mol l^−1^, 98%) were tested. The MLS elicited in response to 0.025% and that to 98% were significantly different. The MSL at all concentrations differed significantly from the control concentration (see Materials and Methods). The number of bees examined is given in each bar. Error bars indicate the 95% confidence interval (CI). The asterisks indicate the intensity levels used for subsequent experiments.

To define the minimum low intensity level, near the threshold for visual and olfactory stimulation in our experimental setup, we trained bees (see ‘Training protocol’, below) using a range of intensity levels (Fig. 2). We defined the minimum level for visual and olfactory learning as the lowest possible magnitude of stimulation that induced a learning performance significantly different from a negative control and from that induced by other higher intensity levels of stimulation. To establish the minimum intensity level for unimodal visual learning, we trained bees to associate a reward (see ‘Training protocol’, below) using light from a monochromatic blue LED (peak λ=458 nm) with intensities that ranged from 0 to 45.7 µmol photons m^−2^ s^−1^ measured with a LI-COR portable spectroradiometer (model Li-1400; Lincoln, NE, USA). We programmed the automatized system (see ‘Experimental setup’, above) to deliver light of five intensities: control (no light), 4.6 µmol photons m^−2^ s^−1^ (10% of maximum intensity), 18.3 µmol photons m^−2^ s^−1^ (40%), 22.9 µmol photons m^−2^ s^−1^ (50%) and 45.7 µmol photons m^−2^ s^−1^ (100%) (Fig. 2).

The median learning score of bees (MLS; see ‘Calculation of response variables’, below) differed across intensity levels (Kruskal–Wallis *H*_4_=33.018, *P*<0.0001). Pairwise comparisons were performed using Dunn's (1964) procedure with a Bonferroni correction. The *post hoc* analysis (Fig. 2A) showed that the MLS elicited by the lowest light intensity level (4.6 µmol photons m^−2^ s^−1^, 10%, mean rank=87.85) differed from that for the medium level stimulus (22.9 µmol photons m^−2^ s^−1^; 50%; mean rank=118.62; *P*=0.035) and from the control (0%; mean rank=42.19; *P*=0.007).

Similarly, to delimit the lower end of olfactory stimulation, we used 1-hexanol 98% (Sigma-Aldrich, H13303) and obtained five different concentrations that varied between 0% and 98% by diluting the pure molecule in mineral oil: control (0%, no odor), 0.0002 mol l^−1^ (0.025%), 0.02 mol l^−1^ (2.5%), 2 mol l^−1^ (25%) and 7.8 mol l^−1^ (98%; undiluted) (Fig. 2B). We found differences in the induced MLS responses across odor concentrations (Kruskal–Wallis *H*_4_=51.825, *P*<0.0001). In particular, the MSL differed between the low concentration (0.025%; 0.0002 mol l^−1^; mean rank=78.91) and the undiluted condition (98%; 7.8 mol l^−1^; mean rank=130.79) (Dunn's, *P*<0.0001), while the MSL elicited at all concentrations differed from the control concentration (Dunn's, *P*=0.0001; Fig. 2B).

After defining minimum intensity levels of visual and olfactory stimulation, we used these in subsequent experiments using unimodal (visual or olfactory) stimuli as well as bimodal stimulation (visual+olfactory) that included middle and higher levels of intensity, aimed to represent a range of saliences (see Fig. 1C). The range for unimodal visual stimulation was as follows: low intensity (4.6 µmol photons m^−2^ s^−1^, 10% of maximum intensity), mid intensity (22.9 µmol photons m^−2^ s^−1^, 50% of maximum intensity) and high (45.7 µmol photons m^−2^ s^−1^, 100% maximum intensity). The range for unimodal olfactory stimulation was determined in this fashion: low concentration (0.0002 mol l^−1^; 0.025%), mid (2 mol l^−1^; 25%) and high (7.8 mol l^−1^; 98%, undiluted). Finally, the bimodal stimulation was provided by the execution of the programmed electronic setup sequence that delivered combinations of simultaneous olfactory and visual stimuli at low (odor 0.025%+blue light 10%), mid (odor 25%+blue light 50%) and high intensities (odor 98%+blue light 100%).

### Experimental settings

We compared the performance of bees trained to unimodal and bimodal stimuli within each of the three intensity levels. Each experimental bee received a single type of stimulation, either unimodal (visual or olfactory) or bimodal, at a single intensity level: low, mid or high. We delivered all combinations of modalities and intensities in a pseudorandom order within each experimental cycle. We applied absolute conditioning where a specific conditioned stimulus (CS+) is associated with a reward. The treatments in our experiment consisted of the application of CS+ using the distinct unimodal (visual/olfactory) or one bimodal (visual+olfactory) stimulus at one of the three possible combinations of intensity levels that we defined.

### Training protocol

We used classical conditioning of the PER (Bitterman et al., 1983; Giurfa and Sandoz, 2012; Hori et al., 2006; Matsumoto et al., 2012; Takeda, 1961). We adapted the original protocol as described by Jernigan et al. (2014). For the acquisition phase, we allowed the bees to acclimate for 15 s before starting the training procedure. A pipette holding a small drop of sucrose–water (|1 μl; 50% w/w) was placed within 1 cm of the chamber entrance during the first 10 s in which the stimulus (CS+) was delivered. During the following 3 s of CS+ stimulation, we paired the CS and the sucrose reward (unconditioned stimulus, US), by gently touching the tip of the antennae to elicit the PER. We allowed the bee to drink the reward for 3 s. Thus, we trained the individuals to associate the CS+ presented for 13 s with the US that overlapped for 3 s. We waited 15 s before turning the rotatory platform to locate the following bee. Each training trial (15 s of acclimation, 13 s of stimulation and the final 15 s period of post-stimulation) was repeated 10 times at intervals of 10 min for each bee (see Fig. 1B). Finally, a memory retention test was conducted after 24 h by exposing the bees to the CS without providing the reward (see Fig. 1B). Individuals were tested for motivation and if a PER was not observed, the bee was removed from the subsequent experiments. For both acquisition and memory retention, we recorded the PER response and latency to exhibit PER.

### Calculation of response variables

We employed the PER directly as a binary dependent variable (1/0) in both the generalized linear and mixed models (see ‘Statistical analysis’, below). In addition, the learning performance of bees at the group level was measured as the percentage of PERs over 10 trials. The latency to elicit a PER response was measured as the time in seconds between the start of the CS presentation and the beginning of a PER. We computed a MLS of each individual bee as the sum of PERs across trials to summarize the dynamics over trials. Following previous work (Riveros and Gronenberg, 2012; Riveros et al., 2020), we computed the average latency response for bees responding in at least three trials.

### Statistical analysis

To explore how the bees' response was affected by the different stimuli modalities and by the manipulation of stimulus intensity, we divided our analysis in two phases: acquisition and memory retention. First, we employed a generalized linear mixed model (GLMM) to study the effect of modality (levels in the model: olfactory, visual and bimodal), intensity (levels in the model: low, mid and high), and the interaction between these factors on the PER response (binary) across 10 training trials during acquisition (10 levels in the model). Then, to study the bees' conditioned PER during the memory retention test, we used a generalized linear model (GLM). We carried out both GLMMs and GLMs in R v.4.0.3 (http://www.R-project.org/) with binomial error distribution using the glmer() function (Bates et al., 2015). These models permit analysis of binary PER data and, in the case of acquisition, allow incorporation of the training trials as within-subjects factors (repeated measures) as well as between-group comparisons (Harrison et al., 2018; Pirk et al., 2013). We used the PER as a dependent factor for the GLMMs and GLMs. In both cases, we introduced modality (3 levels), intensity (3 levels) and trial (10 levels; the repeated measurement component, during acquisition) as independent factors; individual honey bees were included as random factors. We checked on adequate models based on the Akaike information criterion (AIC). To test the effect of individual factors, we used χ^2^ analysis for both GLMMs and GLMs, using the function ‘Anova’ of the *car*() package (Fox and Weisberg, 2019). Then, to determine where significant effects lay across the different levels of factors, we used the package *emmeans*() to obtain pairwise comparisons (Tukey HSD method with Bonferroni correction), estimated marginal means (EMMS), odds ratios and predicted probabilities (https://github.com/rvlenth/emmeans). We also employed GLMMs to test for intensity effects within modalities.

Finally, to study the reaction times of bees during both acquisition and memory retention tests, we employed two-way ANOVA. To analyze the reaction time (s) of conditioned PER during acquisition, we obtained the mean latency across the 10 trials for each individual bee. Therefore, mean latency time was included as dependent variable. Modality, intensity, and the interaction term modality×intensity were considered as independent factors on both analyses.

## RESULTS

We collected and prepared for training 680 bees. We excluded individual bees before the onset of the experiment if they failed a motivation test (a PER after moving the reward towards the bee without touching the antennae and preventing it from drinking) or if they exhibited spontaneous responses to the CS. After the exclusion of bees failing these two criteria (total *n*=232 bees; lack of motivation *n*=223; spontaneous response *n*=9 bees), we conducted experiments employing 448 individuals. The experiments consisted of nine treatments in a fully factorial design with three modalities: olfactory, visual and bimodal, at three levels of intensity (low, mid and high; see Materials and Methods and Fig. 1). We studied the conditioned PER responses of bees across 10 trials during the acquisition phase and 24 h later during the memory retention test.

### Effects of intensity and sensory modality on learning acquisition

**Fig. 3.**
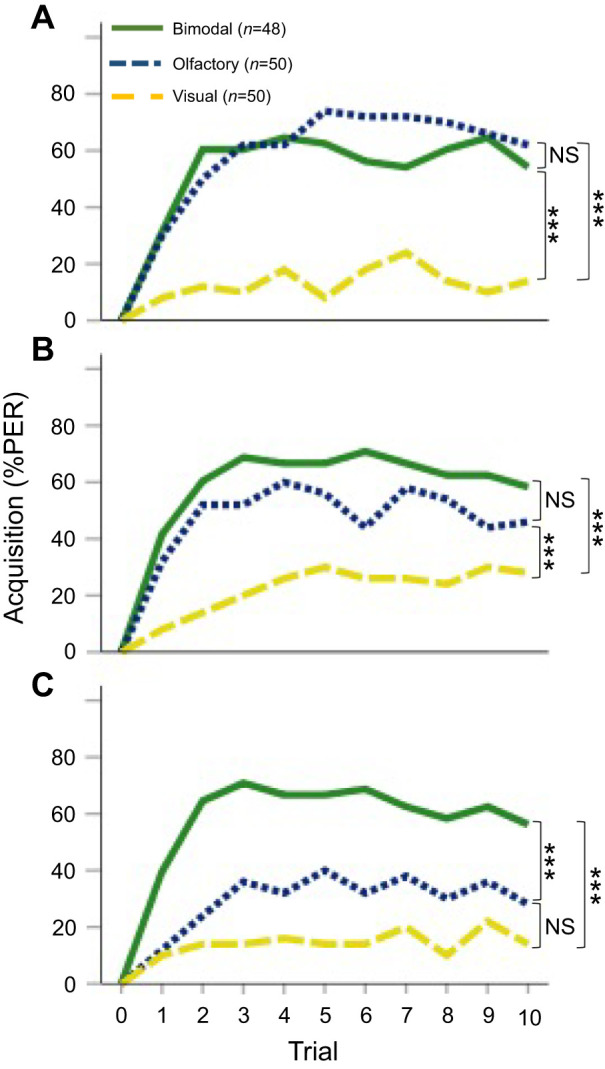
**Learning curves comparing the percentage PER of honey bees during acquisition.** During 10 trials of absolute conditioning, honey bees were trained with one of three different stimuli: unimodal (olfactory or visual) or a bimodal compound (olfactory+visual), at different intensities: (A) high, (B) mid and (C) low (see Materials and Methods). Each bee was trained with a single combination of stimuli. Visual intensity range: low (4.6 µmol photons m^−2^ s^−1^, 10% of maximum intensity), mid (22.9 µmol photons m^−2^ s^−1^, 50% of maximum intensity) and high (45.7 µmol photons m^−2^ s^−1^, 100% maximum intensity). Olfactory stimulation range: low concentration (0.0002 mol l^−1^, 0.025%), mid (2 mol l^−1^, 25%) and high (7.8 mol l^−1^, 98%, undiluted). Bimodal stimulation consisted of combinations of simultaneous olfactory and visual stimuli at low (odor 0.025%+blue light 10%), mid (odor 25%+blue light 50%) and high intensity (odor 98%+blue light 100%). Significance levels are indicated by asterisks (***P<0.0001); ns, no significant difference (see Results).

Bees learned three intensity levels of unimodal (olfactory/visual) and bimodal stimulation, associating them with the reward while showing increasing and differential PER across trials (GLMM: trial effect: χ^2^_1,444_=29.506; *P*<0.0001; Table S1A; Fig. 3). Such differential conditioned associations, measured as changes in the probability of PER, significantly depended upon the specific modality (GLMM: modality effect: χ^2^_2,444_=129.508; *P*<0.0001; Table S1A) and intensity level (GLMM: intensity effect: χ^2^_2,444_=6.891; *P*=0.0305; Table S1A). In consequence, the learning acquisition of bees was affected by the modality and intensity interaction (GLMM: modality×intensity interaction effect: χ^2^_4,444_=19.263; *P*<0.0001; Table S1A).

**Fig. 4.**
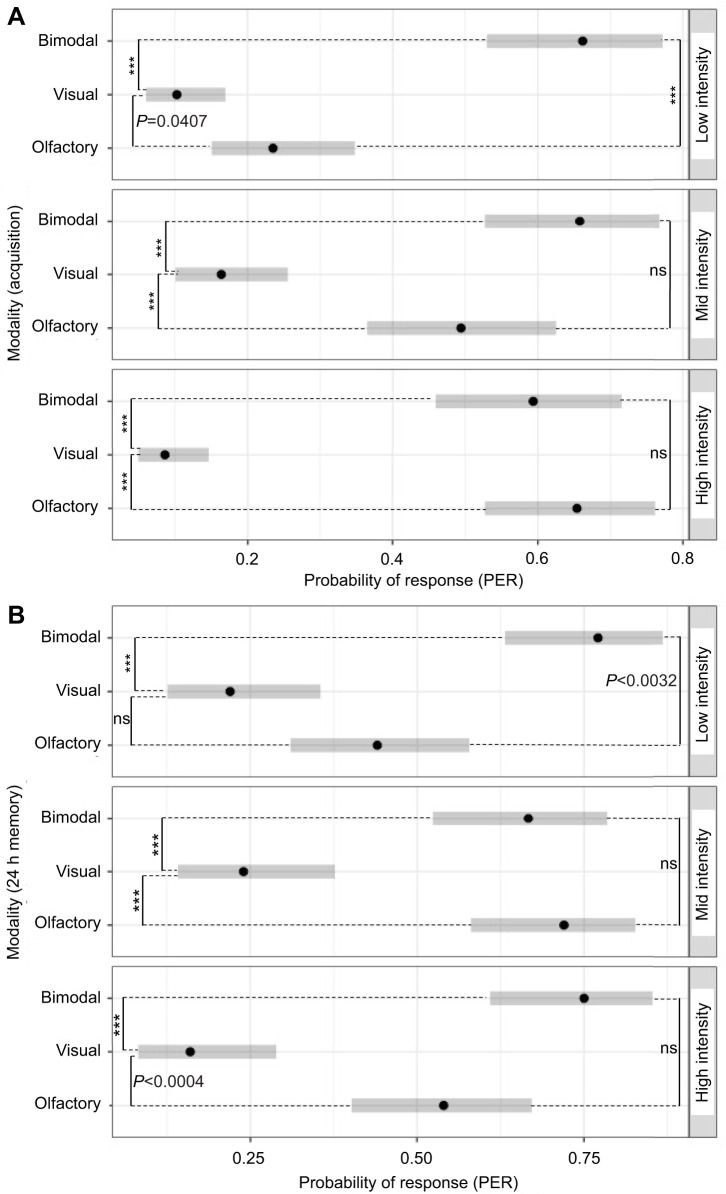
**Predicted probabilities of PER for each modality and intensity level during acquisition and memory retention.** (A) During acquisition at low intensity, bimodal stimulation is predicted to produce an elevated PER, much higher than that induced by both olfaction and visual stimulation. However, at higher intensity, the predicted probability of PER induced by olfactory stimulation increases and becomes indistinguishable from that resulting from bimodal stimulation; at high intensity, the predicted probability of a PER response remains low for visual stimulation. (B) During memory retention, the same pattern of predicted probabilities for PER shown during acquisition remains: the highest enhancement of the PER response in the bimodal relative to unimodal stimulation is produced at low intensity, while at higher intensity, this advantage attenuates. The predicted probability of PER is derived from the *post hoc* analyses for the GLMM and GLM models for acquisition and memory retention, respectively (see Results; Table S1 and Table S2) and was obtained after correcting the number of contrasts by the Tukey method (based on EMM pairwise comparisons; see Table S2). ***P<0.0001; ns, not significant. Error bars represent the 95% CI.

At low stimulus intensity, bees had significantly higher conditioned PER when trained using bimodal stimuli versus unimodal stimuli (*post hoc*, low intensity: olfactory–bimodal, Tukey: *z* ratio=4.654, *P*<0.0001; visual–bimodal, Tukey: *z* ratio=6.953, *P*<0.0001; Fig. 3C; Table S2A). There was no difference between unimodal stimuli at low intensity (Tukey: *z* ratio=2.423, *P*=0.0407; Fig. 3C; Table S2A). At mid and high intensity, there was no difference in acquisition performance between bees trained using unimodal olfactory stimulus conditioning and bimodal stimulus conditioning (mid intensity: olfactory–bimodal, Tukey: *z* ratio=1.735, *P*=1.1923; Fig. 3B; high intensity: olfactory–bimodal, Tukey: *z* ratio=0.667, *P*=0.7824; Fig. 3A; Table S2A). At mid and high intensity, unimodal visual conditioning performance was significantly lower than the other modalities (mid intensity: olfactory–visual, Tukey: *z* ratio=4.073, *P*=0.0001; Fig. 3C; high intensity: olfactory–visual, Tukey: *z* ratio=7.376, *P*=0.0001; Fig. 3A; Table S2A). We display these *post hoc* contrasts for the GLMM model (Table S2a) as predicted probabilities of PER in Fig. 4A.

We examined the effects of modality and intensity on the reaction time of bees. Reaction time during acquisition was affected by the type of modality (two-way ANOVA, *F*_2,334_=14.580, *P<*0.0001) but not by the intensity level (two-way ANOVA, *F*_2,334_=0.538, *P=*0.584). The reaction times elicited by olfactory and bimodal stimulation were the same (Tukey *z* ratio=0.165, *P*=0.766). However, the visual stimulation produced significantly longer latency compared with olfactory and bimodal stimulation (Tukey *z* ratio=1.149, *P*<0.0001; Tukey *z* ratio=1.314, *P*<0.0001, respectively).

### Effects of sensory modality and intensity during the memory retention test

During memory retention, the pattern of conditioned response to unrewarded stimuli was very similar to that elicited during the previous acquisition phase. The type of modality stimulus produced a lasting and differential effect on the conditioned response of bees during the memory test (GLM: modality effect: χ^2^_2,444_=72.2226; *P*<0.00001; Table S1B). At low stimulus intensity, bees showed the greatest relative enhancement in the response. That is, at low stimulus intensity, the conditioned PER response to the bimodal compound was significantly higher than that to both unimodal stimuli (olfactory–bimodal, Tukey: *z* ratio=3.259, *P*=0.003; visual–bimodal, Tukey: *z* ratio=5.119, *P*<0.0001; Table S2B). At low stimulus intensity, there was no difference between unimodal stimuli (olfactory–visual, Tukey: *z* ratio=2.304, *P*=0.055; Table S2B). There was no difference in the bees’ response to olfactory and bimodal stimuli at mid and high intensity (mid, Tukey: *z* ratio=0.572, *P*=0.835; high, Tukey: *z* ratio=2.143, *P*=0.081; Table S2B). At mid and high intensity, the response to visual stimulation was different to both unimodal olfactory (olfactory–visual: mid intensity, Tukey: *z* ratio=4.589, *P*<0.0001; high, Tukey: *z* ratio=3.798, *P*=0.0004; Table S2B) and bimodal stimulation (mid, Tukey: *z* ratio=4.093, *P*<0.0001; high, Tukey: *z* ratio=5.408, *P*<0.0001; Table S2B). The predicted probabilities derived from these *post hoc* procedures for the GLM model for memory retention (Table S2b) are shown in Fig. 4B.

The modality explained the general differences in the latency of bees during the memory retention test (two-way ANOVA, *F*_2,212_=5.207, *P=*0.006). The response of bees to visual stimuli was slower, compared with that to olfactory (Tukey *z* ratio=1.92, *P*=0.039) and bimodal stimuli (Tukey *z* ratio1.92, *P*=0.004). Lastly, the reaction time to olfactory and bimodal stimulation was roughly the same (Tukey *z* ratio=0.44, *P*=0.552).

### Contrasts within modalities across intensities

We also investigated how learning was affected within each modality (visual, olfactory and bimodal) across the levels of intensity during acquisition (Table S3). Olfactory learning was the only modality significantly affected across levels of intensity (GLMM: olfactory intensity level effect: χ^2^_2,150_=21.468; *P*<0.001; trial effect: χ^2^_9,150_=16.527; *P*<0.0001; Table S3b). The responses induced by olfactory stimuli of low intensity were different from the learning achieved with olfactory stimuli at both mid and high intensity (*post hoc*: low–mid, Tukey: *z* ratio=−2.923, *P*=0.0097; low–high, Tukey: *z* ratio=−4.611, *P*<0.001; Table S3b), while responses to mid and high intensity stimuli were quite similar (*post hoc*: mid–high, Tukey: *z* ratio=−1.698, *P*=0.2059). Visual learning was not significantly impacted across different levels of intensity (GLMM: visual intensity level effect: χ^2^_2,150_=3.7962; *P*=0.15; trial effect: χ^2^_9,150_=10.3532; *P*<0.001; Table S3a). Finally, the learning achieved employing bimodal stimulation was unaffected by the different intensity levels (GLMM: bimodal intensity level effect: χ^2^_2,144_=0.6618; *P*=0.718; trial effect: χ^2^_9,144_=4.6291; *P<*0.05; Table S3c).

During memory retention, olfactory learning was again the only modality impacted by intensity level (GLMM: olfactory intensity level effect: χ^2^_2,150_=1.0464; *P*=0.0189; Table S4b); olfactory learning for low and mid intensity was the only significantly different contrast (*post hoc*: low–mid, Tukey: z ratio=−2.792, *P*=0.0145). Visual learning was unaffected across intensities (GLMM: visual intensity level effect: χ^2^_2,150_=1.0464; *P*=0.593; Table S4a). Lastly, bimodal learning during the memory retention test was not impacted differentially across intensities (GLMM: bimodal intensity level effect: χ^2^_2,150_=1.4648; *P*=0.4807; Table S4c).

## DISCUSSION

Our goal was to examine potential interactions between vision and olfaction within bimodal stimuli while inquiring about the role of stimulus intensity during a learning task in harnessed honey bees. We found that relative to its unimodal constituent elements, a multimodal stimulus does not necessarily lead to the highest performance; such a difference in the magnitude of learning depends on the intensity of its constituent unimodal elements. Our results suggest that during bimodal learning and memory, the highest relative enhancement in performance is achieved by employing unimodal components of low intensity. When employing unisensory stimuli at relatively low intensity and combining them for bimodal conditioning, we found the bees achieved significantly higher learning performance. However, at higher intensity, the relative advantage of the bimodal condition in terms of learning performance diminished while, simultaneously, the olfactory component showed a higher performance. This might not be surprising, as, from a purely informational perspective, multimodal signals may not necessarily be more advantageous than unimodal signals (Rubi and Stephens, 2016b; Wilson et al., 2013). At the perceptual level, physical properties of the unimodal components within a bimodal signal interact, thus enhancing or reducing the response and resulting in processing benefits (Stein and Stanford, 2008). Signal intensity may determine those benefits and, hence, it may be important during unimodal and bimodal learning and memory. The synergistic effects of the unimodal components during bimodal stimulation are relevant, especially at low intensity.

Similar near-threshold situations have been reported in humans and other animals. For instance, the so-called ‘cocktail party problem’ describes a noisy context where visual input may aid in understanding the voice of an interlocutor (Bee, 2015; Kayser et al., 2011). Despite being initially approached as a unimodal phenomenon (i.e. auditory scenes analysis) (Bee, 2015), the cocktail party problem is a well-known scenario that illustrates multisensory integration (Ross et al., 2007). In essence, the visual information about lip movements enhances the perception of the auditory signal (Kayser et al., 2011). Previous work described a neuronal substrate for an analogous ‘flower party effect’ in honey bees (Strube-Bloss and Rössler, 2018). Likewise, our results support the idea of an interaction between visual and olfactory information that enhances learning and memory at near-threshold intensities, the pattern behind a flower party effect. However, our results also agree with a derived prediction of the cocktail party effect: when a single modality is strong enough to surpass the threshold for masking interference (noise), no other additional modality should be required for effective communication. That is, at higher intensities, when unisensory stimuli elicit stronger responses, the processing of two unisensory inputs is more likely to be redundant, thereby reducing the need for multisensory integration (Ross et al., 2007).

We interpret the results of our experiment with restrained bees to be like a flower party effect, where the multisensory benefits are dependent on the salience of the unimodal components. In real-world situations, under what circumstances might a flower party effect be encountered by bees? Typically, flowers emit complex signals (Hebets and Papaj, 2005), and pollinators tend to find specific plant hosts more efficiently through multimodal signals (Burger et al., 2010; Dötterl et al., 2014). However, during transmission, flowers' visual and olfactory signals are degraded by several environmental factors (e.g. cloud cover, temperature, humidity, wind, etc.), affecting signal transmission parameters (visual: medium absorption, scattering and filtering; olfactory: distance, wind turbulence) (Bradbury and Vehrencamp, 1998). Therefore, during foraging, bees might experience environmental conditions that influence the conspicuousness of flower multimodal signals. This degradation is also the basis of competing flowering signals, concealing floral displays (Leonard et al., 2011a,b,c). Here, the associative learning ability of bees allows floral constancy – the short-term specialization of pollinators on flower type – depending on the relationship between floral rewards and signals (Schiestl and Johnson, 2013). Once such floral constancy is established, bees should integrate floral displays, including near-threshold multimodal signals, to access rewards.

Even when assuming interpretations from information theory where multimodal signals do not offer additional information per se (Rubi and Stephens, 2016b; Wilson et al., 2013), the interactions between the intensities of the elements of a composed signal might enhance its detection and/or processing (Hebets and Papaj, 2005; Leonard et al., 2011c; Solvi et al., 2020), resulting in improved learning (Katzenberger et al., 2013; Mackintosh, 1974; Rescorla and Wagner, 1972). We suggest that during multimodal learning of harnessed bees, a few combinations of functional interactions (Leonard et al., 2011c; Raguso, 2004) might occur, depending on the intensity of its components. Our finding of a bimodal enhancement in learning performance after combining near-threshold unimodal visual and olfactory stimuli might be considered ‘synergistic’ (Raguso, 2004; Raguso and Willis, 2002), because the combined signal allowed a high bimodal associative learning, while its elements resulted in a low learning performance on their own. In contrast, although when employing mid and high intensity stimulus levels, the relative bimodal advantage decreased, the combined bimodal stimuli still elicited a high learning performance. In addition, at these higher intensities, olfactory stimulation induced even higher learning performance than that elicited by the bimodal stimuli. Here, the effectiveness of a bimodal signal might be dominated by olfaction alone, while visual stimulation might be of secondary influence. Therefore, we propose that, at near-threshold stimulus intensities, compound signals eliciting a behavioral response deploy synergistic interactions, while only at relatively high intensities might the elements of a multimodal signal be considered either ‘complementary’ or ‘redundant’ (Leonard et al., 2011c; Raguso, 2004; Raguso and Willis, 2002). Our data, therefore, seem to support a set of explanations within the ‘efficacy-based hypothesis’ (multimodal components increase effective transmission, detection or signal processing by the receptor) for the establishment of multimodal signals in communication systems in general and in pollination systems in particular (Hebets and Papaj, 2005; Leonard et al., 2011c). Thus, at low stimulus intensities, not only does the idea of a flower party effect fit nicely into the framework where the floral complexity of multimodal signals facilitates detection against background noise (detection-based hypothesis; Chittka and Spaethe, 2007; Leonard et al., 2011c) but also a multicomponent signal is beneficial because it might allow parallel rather than serial processing (signal-processing hypothesis; Hebets and Papaj, 2005; Leonard et al., 2011c).

Indeed, at the neural level, a parallel architecture is characteristic of the mushroom bodies (MB), a region in the insect brain involved in the processing and integration of multimodal information, learning and memory (Ehmer and Gronenberg, 2002; Erber, 1978; Gronenberg, 2001; Homberg and Erber, 1979; Menzel and Giurfa, 2001). In honey bees, MB output neurons exhibit cross-modal integration after unimodal and bimodal stimulation (Strube-Bloss and Rössler, 2018). These output neurons categorize its responses to visual, olfactory and bimodal stimuli. Remarkably, a neuronal enhancement of olfactory and visual input was detected when presented as a compound (Strube-Bloss and Rössler, 2018). Our behavioral results might expand this neural circuit perspective towards the modulation of associative learning and memory of bimodal compounds.

Together, these results of cross-modal interactions are related and agree with the narrative of the principles of multimodal integration, postulated after recordings of unimodal and multimodal cells of the superior colliculus of cats (Meredith and Stein, 1983, 1996; Stein and Stanford, 2008). To be effectively integrated by the brain as a multimodal signal, the unimodal elements require some correspondence in the temporal and spatial domains, the first and second ‘rules’, respectively (Otto et al., 2013; Stein and Stanford, 2008). The third principle, termed inverse effectiveness, states that two or more sensory stimuli produce a maximal multisensory response enhancement when the unisensory stimuli are minimally effective in evoking responses (Alvarado et al., 2007; Chandrasekaran, 2017; Holmes, 2009; Stanford and Stein, 2007; Stein and Stanford, 2008). Importantly, when the unisensory stimuli are emitted at high intensities, they evoke stronger responses by themselves, providing redundant information, and reducing the need for or the importance of integrating different modalities (Otto et al., 2013; Ross et al., 2007; Stein and Stanford, 2008). We argue that our results are analogous to this principle. Here, we report that the principle of inverse effectiveness may act during multisensory tasks that involve learning and memory in honey bees.

Our main goal was to compare the learning performance of unimodal (olfactory, visual) and bimodal stimulation within each level of intensity presented (low, mid and high). Our results show that the efficacy of bimodal learning performance is relative to the intensity of its unimodal components. A secondary comparison of natural interest was to contrast learning within modalities across intensities. Interestingly, only olfactory learning was significantly impacted by intensity when comparing within modalities across intensities, during acquisition and memory retention (see Results; Fig. 4; Tables S1 and S2). Hence, we found that bimodal stimuli are capable of inducing high learning performance across intensity levels (low, mid or high). Our results indicate that bimodal stimuli retain their associative strength across a wide spectrum of signal-to-noise ratios, from acquisition to memory. Nonetheless, this occurrence with unimodal constituent elements of low salience reflects the modulating effect of intensity during bimodal stimulation, similar to the findings of research in vertebrate systems (Meredith and Stein, 1983, 1996; Stein et al., 1988).

Our results also confirm that the efficacy of olfactory stimulation is significantly impacted by intensity level. This is consistent with previous work showing that odorant intensity correlates with improved performance during learning and discrimination tasks, as a result of the codification strategy of the olfactory system (Leonard and Masek, 2014; Wright and Smith, 2004; Wright et al., 2009). In contrast to olfactory learning, the influence of visual stimulation remained both low and mostly unaffected across intensity (see Results; Fig. 4). The reaction pattern of bees shows that visual stimulation at low and high intensity tends to induce an even lower response, relative to visual stimulation at mid intensity. In general, this pattern appears consistent with the threshold test for visual learning (Fig. 2) and also during acquisition and memory retention (Figs 3 and 4). Such response patterns were presented at a higher variance during memory retention tests (Fig. 4B), which might be interpreted as a consequence of an overall weakening of the association strength. As in many other visual systems, visual performance in honey bees declines at low light intensity (Warrant et al., 1996); furthermore, this, the response of trained bees also declines at high light intensity (Menzel, 1981; Rose and Menzel, 1981). This bright light effect is explained as the result of the specific response function of the lamina monopolar cells (Menzel, 1981). Our results might therefore be consistent with these accounts where the visual perception of bees is less acute at both low and high intensity levels (the dim and bright light effects, respectively). We argue that the synergistic interaction between visual and olfactory stimulation is modulated by their intensity and is critical in shaping the relative associative strength of the bimodal stimulus. Our results highlight this when both visual and olfactory low intensity stimuli are combined, merging into bimodal stimuli of high associative strength despite their low intensity (Figs 3 and 4). When unimodal components of higher intensity are combined, olfaction takes a leading role in the bimodal condition.

Assessing the effect of the intensity of the elements that comprise stimuli during multimodal learning might explain some discrepancies regarding the beneficial nature of multimodal signals. Our results suggest that the benefits of multimodal stimuli might depend on the intensity of the unimodal components. Therefore, multimodal signals are not necessarily better than unimodal ones. Consequently, the intensity of the individual elements should be considered when examining the effectiveness of a multimodal signal in producing synergistic effects during processing even after recognizing intrinsic differences between free-flight and restrained methods (Jernigan et al., 2014; Kulahci et al., 2008; Leonard and Masek, 2014; Leonard et al., 2011b,c; Riveros et al., 2020).

Finally, a speeding up of responses is predicted to be one the advantages provided by multimodal stimuli (Hebets and Papaj, 2005; Leonard and Masek, 2014; Leonard et al., 2011c). Moreover, physiological evidence in mammals and computational models point to benefits in terms of reaction times as a multimodal effect (Chandrasekaran et al., 2011; Colonius and Diederich, 2017). Despite this, we found that reaction times did not conform with the principle of inverse effectiveness, nor did we found a consistent reduction in latency after employing bimodal stimuli, as shown previously (Riveros et al., 2020). However, the acceleration in response time is predicted to occur only when the elements of a multimodal stimulus elicit similar performance levels; that is, the speeding up of the latency should be largest after controlling the salience of unimodal stimuli, ensuring the induction of similar effectiveness (Otto et al., 2013). Further research may address the speeding up of PER during the multimodal learning task, controlling for unisensory stimuli leading to equivalent performance.

### Conclusion

In conclusion, our data suggest that the performance benefits associated with the use of a bimodal signal during learning and memory tasks are dependent on the interaction of its components with their intensity. Specifically, visual and olfactory stimuli that independently elicit low performance, when bimodally combined, produce a significant enhancement during both acquisition and memory. This finding, together with the finding that such a magnitude of bimodal enhancements was not present at mid and high intensities, suggests that honey bees integrate bimodal information following the principle of inverse effectiveness during learning and memory. Such integration relies upon neuronal computations occurring when visual and olfactory inputs appear as a compound. The intensity modulation of the components of a bimodal signal would enable honey bees to acquire, retain and respond effectively to changing environmental conditions where bimodal processing is not always the most efficient way to gather useful information. Therefore, the benefits for receivers derived from the integration of multimodal signals result from a fine-tuned relationship between perception mechanisms, cognitive bias and changing physical conditions across environmental contexts.

## Supplementary Material

Supplementary information
