# Supplementary material for: Honey bees respond to multimodal stimuli following the principle of inverse effectiveness
Source: J Exp Biol. 2022 May 24;225(10):jeb243832. doi: 10.1242/jeb.243832 (PMC9206449; doi:10.1242/jeb.243832)
Supplement: Supplementary information [file jexbio-225-243832-s1.pdf]

Journal of Experimental Biology • Supplementary information

Table S1. A) Summary of the binomial GLMM model for the PER in response to unimodal (olfactory / visual) and bimodal stimuli that varied in intensity (low, mid high) across 10 trials during learning (Model fit: link function (logit), conditional R^2= 0.58, AIC = 4446.34, ICC = 0.48, individual honeybees denoted as random effects); **B)** summarizes the binomial GLM model during the a single memory retention test, 24h later (Model fit: χ^2 (8) = 100.09, p = 0.00, link function (logit); conditional R^2= 0.27, AIC =533.41). For both models, N = 444. Significance values are indicated.

a) GLMM Acquisition Model

=PER response ~ Modality X Intensity + Trial + (1 | individual honeybee)

**Coefficients**	**Estimate**	**SE**	**z val.**	***p***	
Intercept	-1.591	0.292	-5.453	<0.0001	
Visual	-0.989	0.408	-2.423	0.015	
Bimodal	1.850	0.398	4.654	<0.0001	
Mid intensity	1.157	0.391	2.957	<0.001	
High intensity	1.816	0.389	4.667	<0.0001	
Trial	0.075	0.014	5.432	<0.0001	
Visual *X* Mid intensity	-0.618	0.568	-1.089	0.276	
Bimodal *X* Mid intensity	-1.175	0.556	-2.113	0.035	
Visual *X* High intensity	-2.010	0.576	-3.490	<0.0001	
Bimodal *X* High intensity	-2.107	0.554	-3.804	<0.0001	
**b) GLM Memory retention Model** =					
=PER response ~ Modality X Intensity					
Intercept	-0.2412	0.2849	-0.846	0.3973	
Visual	-1.0245	0.4447	-2.304	0.0212	
Bimodal	1.4542	0.4462	3.259	0.0011	
Mid intensity	1.1856	0.4247	2.792	0.0052	
High intensity	0.4015	0.4021	0.999	0.3180	
Visual *X* Mid intensity	-1.0726	0.6376	-1.682	0.0925	.
Bimodal *X* Mid intensity	-1.7055	0.6261	-2.724	0.0065	
Visual *X* High intensity	-0.7941	0.6535	-1.215	0.2243	
Bimodal *X* High intensity	-0.5159	0.6251	-0.825	0.4092	

Table S2. Summary of pairwise comparisons contrasting the Modalities (Olfactory, Visual and Bimodal) across Intensities (Low, Mid and High) for **A)** the GLMM model for the bee's PER response during acquisition and **B)** after the GLM model for the bee's PER response during the memory retention phase. For both models, interaction contrasts of model fixed factors. Results are given on the logit (not the response) scale. Confidence level used: 0.95. Results given on the log odds ratio scale; *p* values obtained using the Tukey HSD method.

**Intensity**	**Modality contrast**	**Odds ratio (SE)**	**Estimate (SE)**	**Z. ratio**	***P***
**A)**		**PER Acquisition**			
**Low**	Olfactory - Visual	2.69 (1.10)	0.989 (0.408)	2.423	0.0407
	Bimodal - Olfactory	**6.36** (2.53)	1.85 (0.398)	4.654	<0.0001
	Bimodal - Visual	**17.1** (6.98)	2.839 (0.408)	6.953	<0.0001
**Mid**	Olfactory - Visual	**4.99** (1.97)	1.607 (0.395)	4.073	<0.0001
	Bimodal - Olfactory	1.96 (0.76)	0.675 (0.389)	1.735	0.1923
	Bimodal - Visual	**9.8** (3.90)	2.282 (0.398)	5.729	<0.0001
**High**	Olfactory - Visual	**20.07** (8.16)	2.999 (0.407)	7.376	<0.0001
	Olfactory - Bimodal	1.29 (0.50)	0.257 (0.386)	0.667	0.7824
	Bimodal - Visual	**15.52** (6.38)	2.742 (0.411)	6.664	<0.0001
**B)**		**PER 24h Retention**			
**Low**	Olfactory - Visual	2.79 (1.24)	1.025 (0.445)	2.304	0.055
	Bimodal - Olfactory	**4.28** (1.91)	1.454 (0.446)	3.259	0.003
	Bimodal - Visual	**11.93** (5.77)	2.479 (0.484)	5.119	<0.0001
**Mid**	Olfactory - Visual	**8.14** (3.72)	2.097 (0.457)	4.589	<0.0001
	Olfactory -Bimodal	1.29 (0.56)	0.251 (0.439)	0.572	0.835
	Bimodal - Visual	**6.33** (2.86)	1.846 (0.451)	4.093	0.0001
**High**	Olfactory - Visual	**6.16** (2.95)	1.819 (0.479)	3.798	0.0004
	Bimodal - Olfactory	2.56 (1.12)	0.938 (0.438)	2.143	0.081
	Bimodal - Visual	**15.75** (8.03)	2.757 (0.51)	5.408	<0.0001

Journal of Experimental Biology • Supplementary information

Table S3. Summary of the GLMMs models comparing the effect on the PER performance between intensities (low, mid, and high) within each modality of stimulation (olfactory, visual, and bimodal) during the acquisition phase of the experiments.

a) GLMM Model comparing Visual intensities during acquisition

=PER response ~ Visual low + Visual mid+ Visual high + Trial + (1 | individual honeybee)

Coefficients	Estimate	SE	z val.	*p*
Intercept (Visual low)	-2.626	0.323	-8.136	<0.0001
Visual mid	0.543	0.386	1.407	0.160
Visual high	-0.183	0.399	-0.459	0.646
Trial	0.088	0.027	3.218	<0.001

b) GLMM Model comparing Olfactory intensities during acquisition

=PER response ~ Olfactory low + Olfactory mid + Olfactory high + Trial + (1 | individual honeybee)

Coefficients	Estimate	SE	z val.	*p*
Intercept (Olfactory low)	-1.695	0.315	-5.382	<0.0001
Olfactory mid	1.170	0.400	2.923	<0.01
Olfactory high	1.835	0.398	4.611	<0.0001
Trial	0.091	0.022	4.065	<0.0001

c) GLMM Model comparing Bimodal intensities during acquisition

=PER response ~ Bimodal low + Bimodal mid + Bimodal high +Trial +(1|individual honeybee)

Coefficients	Estimate	SE	z val.	*p*
Intercept (Bimodal low)	0.405	0.313	1.291	0.197
Bimodal mid	-0.020	0.405	-0.050	0.960
Bimodal high	-0.293	0.404	-0.725	0.469
Trial	0.049	0.023	2.152	0.031

Journal of Experimental Biology • Supplementary information

Table S4. GLMM model comparing within modalities and across intensities during the memory retention test.

Coefficients	Estimate	SE	z val.	*p*
**a) GLMM Model comparing Visual intensities during memory retention**				
=PER response ~ Visual low + Visual mid+ Visual high + (1 | individual honeybee)				
Intercept (Visual low)	-1.266	0.341	-3.708	<0.001
Visual mid	0.113	0.476	0.238	0.812
Visual high	-0.393	0.515	-0.762	0.446
**b) GLMM Model comparing Olfactory intensities during memory retention**				
=PER response ~ Olfactory low + Olfactory mid + Olfactory high + (1 | individual honeybee)				
Intercept (Olfactory low)	-0.241	0.285	-0.846	0.397
Olfactory mid	1.186	0.425	2.792	0.005
Olfactory high	0.402	0.402	0.999	0.318
**c) GLMM Model comparing Bimodal intensities during memory retention**				
=PER response ~ Bimodal low + Bimodal mid + Bimodal high + (1|individual honeybee)				
Intercept (Bimodal low)	1.213	0.343	3.532	<0.001
Bimodal mid	-0.520	0.460	-1.130	0.258
Bimodal high	-0.114	0.479	-0.239	0.811
